# Gene-Specific Genetic Complementation between *Brca1* and *Cobra1* During Mouse Mammary Gland Development

**DOI:** 10.1038/s41598-018-21044-2

**Published:** 2018-02-09

**Authors:** Huai-Chin Chiang, Xiaowen Zhang, Xiayan Zhao, Chi Zhang, Jerry Chen, Paula Garza, Sabrina Smith, Thomas Ludwig, Richard J. Baer, Rong Li, Yanfen Hu

**Affiliations:** 1 Department of Molecular Medicine, University of Texas Health San Antonio, San Antonio, TX 78229 USA; 2 Xiangya School of Medicine, Central South University, Changsha, Hunan China; 3 Department of Cancer Biology and Genetics, Ohio State University, Columbus, OH 43210 USA; 4 Department of Pathology & Cell Biology, New York, NY 10032 USA

## Abstract

Germ-line mutations in breast cancer susceptibility gene, *BRCA1*, result in familial predisposition to breast and ovarian cancers. The BRCA1 protein has multiple functional domains that interact with a variety of proteins in multiple cellular processes. Understanding the biological consequences of BRCA1 interactions with its binding partners is important for elucidating its tissue-specific tumor suppression function. The Cofactor of BRCA1 (COBRA1) is a BRCA1-binding protein that, as a component of negative elongation factor (NELF), regulates RNA polymerase II pausing during transcription elongation. We recently identified a genetic interaction between mouse *Brca1* and *Cobra1* that antagonistically regulates mammary gland development. However, it remains unclear which of the myriad functions of *Brca1* are required for its genetic interaction with *Cobra1*. Here, we show that, unlike deletion of *Brca1* exon 11, separation-of-function mutations that abrogate either the E3 ligase activity of its RING domain or the phospho-recognition property of its BRCT domain are not sufficient to rescue the mammary developmental defects in *Cobra1* knockout mice. Furthermore, deletion of mouse *Palb2*, another breast cancer susceptibility gene with functional similarities to *BRCA1*, does not rescue *Cobra1* knockout-associated mammary defects. Thus, the *Brca1*/*Cobra1* genetic interaction is both domain- and gene-specific in the context of mammary gland development.

## Introduction

**Figure 1:**
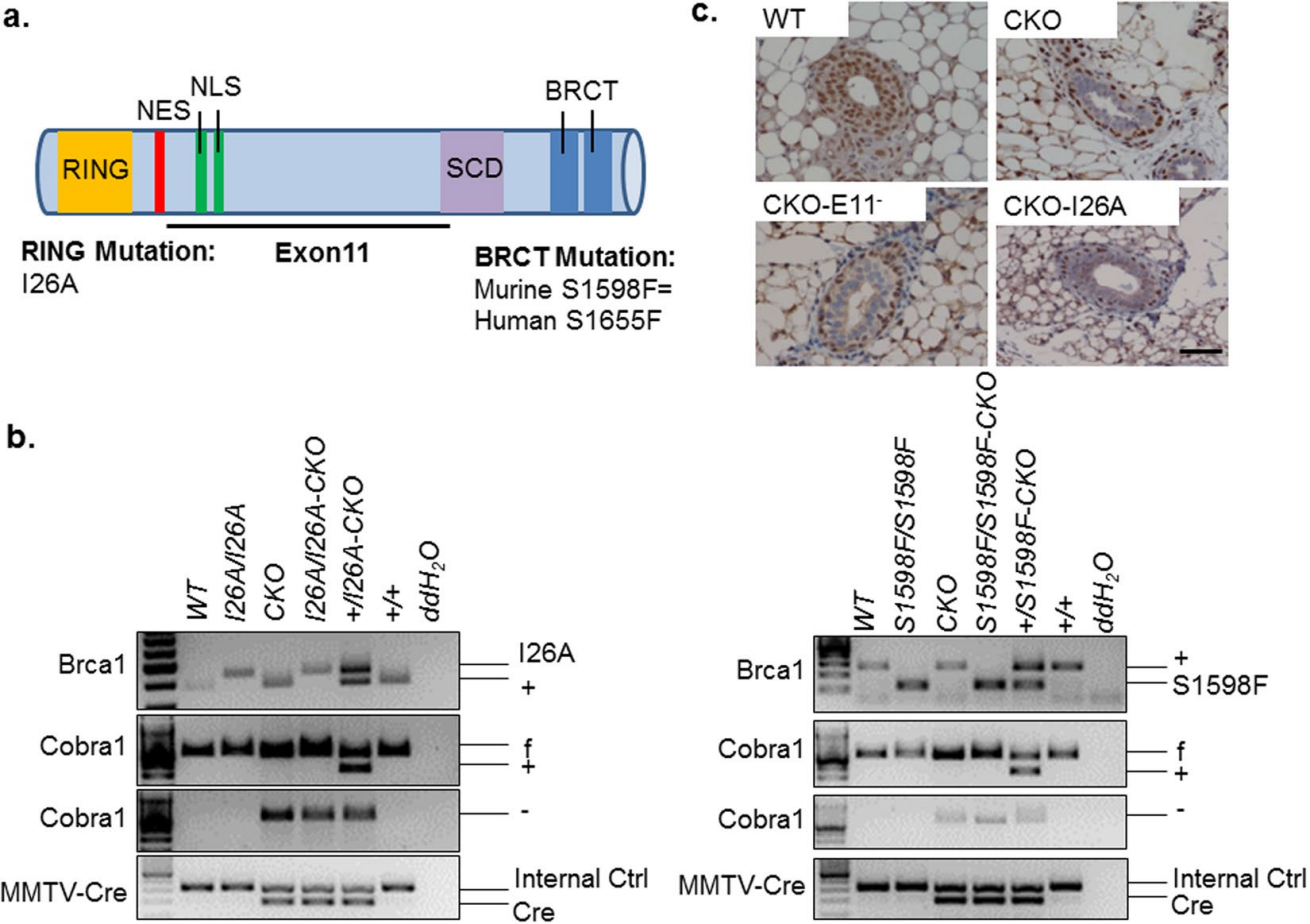
*Brca1* knock-in mouse model for the RING and BRCT mutations. (**a**) Key knock-in mutations in domain structures of BRCA1 protein. Different colors are used to show the structures with RING domain in orange, nuclear export signal (NES) in red, the tandem nuclear localization signals (NLS) in green, the serine cluster domain (SCD) in purple, and two BRCT domains in blue. I26A: isoleucine to alanine at the 26 amino acid position. S1598F: serine to phenylalanine at the 1598 amino acid position. (**b**) Validation of different mutant mice by genotyping. Full-length gels of the PCR analysis are presented in Supplementary Fig. 1. (**c**) COBRA1 immunohistochemistry analysis in mammary gland of 8-week virgin mice. Representative results from at least 4 sets of animals. Scale bar = 50 µM.

Women who harbor germline mutations of *BRCA1* have an increased lifetime risk of developing breast and ovarian cancers^1^. The BRCA1 protein contains multiple functional domains (Fig. 1a), including an N-terminal RING domain, a central region encoded by exons 11–13, and a C-terminal BRCT domain^2^. The RING domain of BRCA1, together with its interacting partner BARD1, constitutes a potent ubiquitin E3 ligase^3–5^. Exons 11–13 encode multiple protein-binding sites^6–9^, including a coiled-coil domain that interacts with the product of the *PALB2* breast cancer susceptibility gene^10–12^, allowing assembly of a BRCA1/PALB2/BRCA2 protein complex that can recruit RAD51 to the sites of DNA double strand breaks (DSBs) and thereby promote DSB repair by homologous recombination (HR)^13,14^. The two BRCT repeats of BRCA1 are capable of recognizing the phosphorylated isoforms of several important repair proteins, thus forming multiple distinct protein complexes that facilitate the DNA damage response (DDR) and DSB repair by HR^8,15–21^. In addition, BRCA1 has been implicated in RNA transcriptional regulation through association with RNA polymerase II (Pol II)^7,22^ and the Cofactor of BRCA1 (COBRA1)^23^, which is identical to the B subunit of the negative elongation factor complex (NELF-B)^24–27^. Despite these advances^19,28–30^, it remains challenging to connect individual functional domains of BRCA1 to specific BRCA1 functions *in vivo*. Aside from the extensive cell culture-based research on BRCA1 functions, recent studies of mouse strains bearing separation-of-function mutations have shed light on the structural-functional relationship in BRCA1 biology^31–35^. For example, using point mutations that separately disrupt the RING and BRCT domains, it was shown that some functions of the latter (BRCT phospho-recognition) but not the former (E3 ligase activity) are essential for BRCA1-mediated tumor suppression^35^.

Using mammary epithelial-specific knockout (KO) mouse models for *Brca1* and *Cobra1*, we recently demonstrated genetic complementation between these two genes during normal mammary gland development and tumor suppression^36,37^. Tissue-specific deletion of *Cobra1* blocked ductal morphogenesis and alveologenesis, demonstrating a crucial role of COBRA1/NELF-B in adult tissue development. Of note, these resulting developmental defects of *Cobra1* ablation were largely rescued by the loss of full-length BRCA1 expression through deletion of *Brca1* exon 11^37^. Reciprocally, *Cobra1* deletion reduced mammary tumorigenesis associated with *Brca1* inactivation^37^. We further showed that the functional antagonism between *Brca1* and *Cobra1* in mammary gland development and tumorigenesis is independent of the role of BRCA1 in HR repair^36,37^. While our published study provides compelling evidence for a functional link between BRCA1 and transcriptional regulation that dictates the developmental outcome in mammary epithelium, it remains unclear whether the ability of genetic complementation observed with the exon 11 deletion mutant of *Brca1* extends to mouse strains carrying other *Brca1* mutations or mutations in other genes functionally related to *Brca1*. Here we address this important question by examining genetic interactions between *Brca1* and *Cobra1* in mice bearing separation-of-function mutations in either the RING (I26A) or BRCT domains (S1598F) of *Brca1*^35^, or a conditional-null mutation of the *Palb2* gene^38^.

## Results

We previously reported that deletion of exon 11 in *Brca1* (BKO or E11^−^) rescued the mammary developmental defect associated with *Cobra1* knockout (CKO)^37^. To discern the contributions of the different functional domains of BRCA1 to its ability to genetically complement *Cobra1* inactivation, we utilized two available knock-in (*KI*) mouse strains, I26A and S1598F, in which the corresponding point mutations disrupt the E3 ligase activity of the RING domain and the phospho-recognition property of the BRCT domain, respectively^35^ (Fig. 1a). Through a series of breeding with CKO, we generated compound mice of *Cobra1*^*f/f*^ *Brca1*^*I26A/I26A*^; *MMTV-Cre* (CKO-I26A) and *Cobra1*^*f/f*^ *Brca1*^*S1598F/S1598F*^; *MMTV-Cre* (CKO-S1598F). Genotyping confirmed the deletion of *Cobra1* and the presence of the desired *Brca1* KI mutations in the compound mutant mice (Fig. 1b) (see Supplementary Fig. 1). Furthermore, we used immunohistochemistry to verify that depletion of COBRA1 protein levels was equally effective in mammary epithelial cells of *Cobra1* KO alone (CKO) and *Cobra1*/*Brca1* compound-mutant (CKO-E11^−^ and CKO-I26A) mice (Fig. 1c) (see Supplementary Fig. 2). Thus, the *Brca1* point mutations did not affect the efficiency of Cre-mediated genetic ablation of *Cobra1*. Of note, breeding for compound mice was challenged by the male sterility of the two *Brca1* KI mutant strains^35^ and by the inability of CKO dams to nurse^37^.

**Figure 2:**
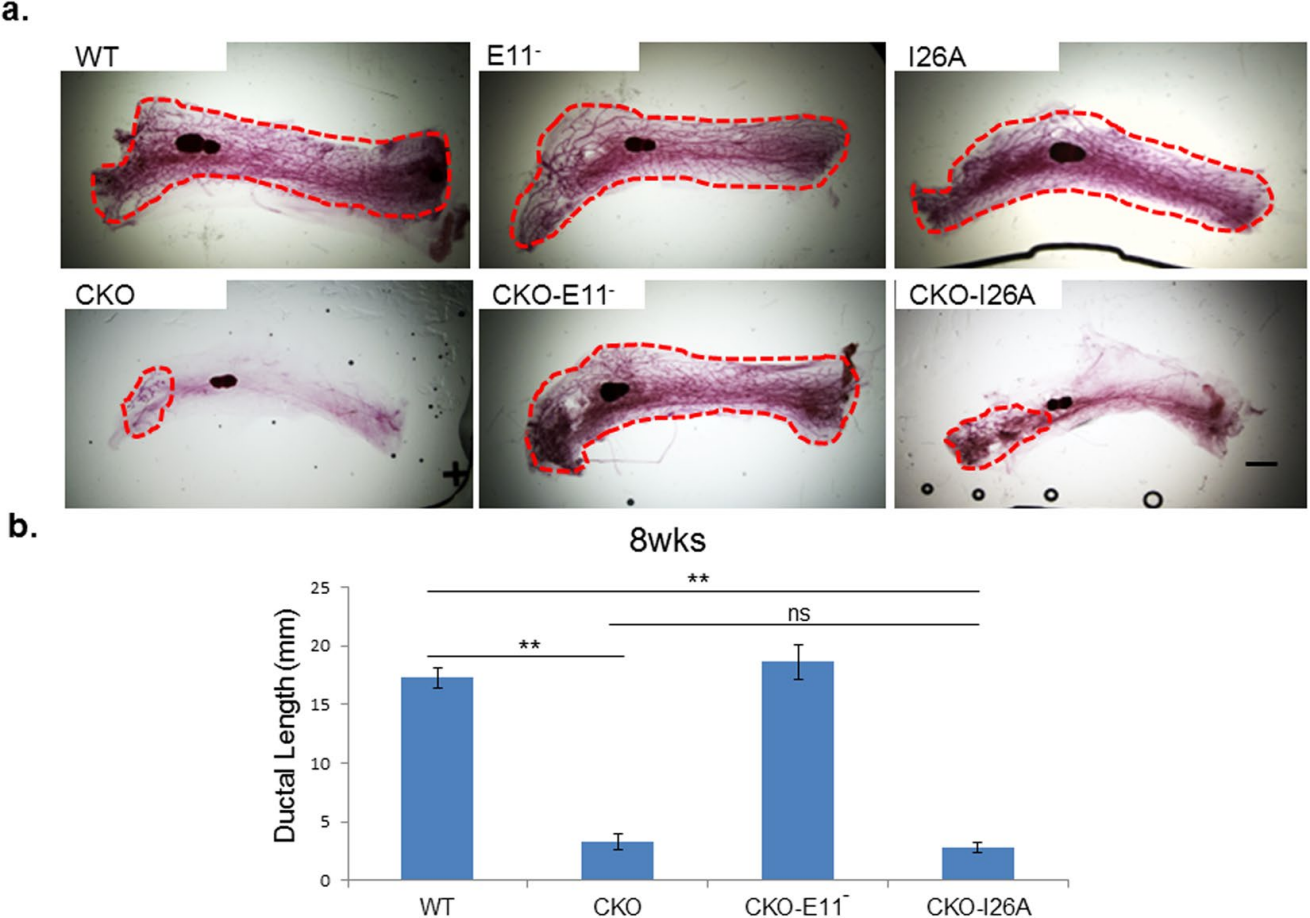
*Brca1* I26A mutation is not sufficient for rescuing the developmental defects of *Cobra1*-KO mammary glands. (**a**) Whole mounts of mammary glands from 8-week virgin mice. Red dash line highlights the boundary of the ductal area. Images are representatives of at least 4 animals in each group. Scale bars = 1 mm. (**b**) Measurement of average ductal length of 8-week mammary glands. The numbers of animals used are: WT = 4, CKO = 6, CKO-E11^−^ = 7, CKO-I26A = 4. Error bars represent s.e.m. Student’s *t*-test was used to calculate P value. **P < 0.01, ns: not significant.

**Figure 3:**
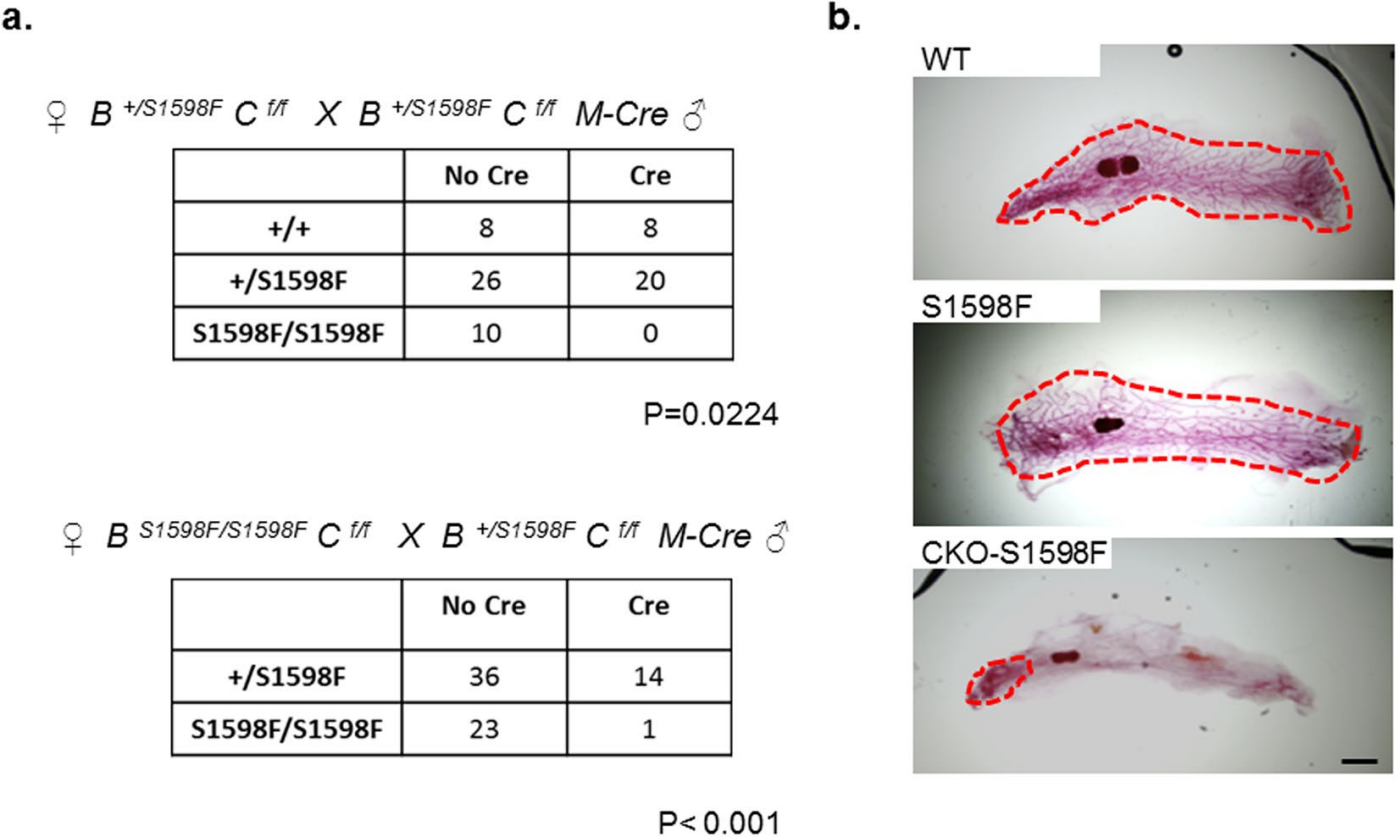
Generation of CKO-S1598F compound mutant mice. (**a**) Chi-square analysis for female progenies obtained from the breeding strategies indicated. The two-tailed P values are calculated by Chi-square goodness-of-fit test with 5 degrees of freedom. (**b**) Whole mounts of mammary glands from 8-week virgin mice. Red dash line highlights the boundary of the ductal area. Scale bars = 1 mm. Mammary ductal growth of a sole surviving CKO-S1598F female mouse is shown.

Mammary ductal growth of the two parental homozygous *Brca1* KI mutant mouse strains (I26A and S1598F) was comparable to that of their WT littermate controls (Figs 2a and 3b). In contrast, age-matched homozygous compound-mutant mice with I26A and *Cobra1* KO (CKO-I26A) exhibited ductal developmental defects as severe as those observed in CKO, as illustrated by both analyses of whole mounts (Fig. 2a) (see Supplementary Fig. 3) and quantification of ductal lengths (Fig. 2b). Thus, unlike *Brca1* exon 11 deletion (E11^−^), the I26A mutation does not rescue the developmental phenotype of CKO mice. Despite extensive breeding, we were only able to generate one female CKO-S1598F compound mutant mouse, a frequency significantly lower than the expected Mendelian ratio (Fig. 3a). We suspect that this could be due to the known leakiness of MMTV-Cre in other tissues and embryonic lethality of the combined *Cobra1* KO and S1598F KI mutation. Of note, the sole surviving female compound-mutant mouse (CKO-S1598F) display no signs of genetic rescue of the mammary defects (Fig. 3b). Taken together, our results indicate that *Brca1* exhibits an allele-dependent genetic interaction with *Cobra1* during mammary gland development.

**Figure 4:**
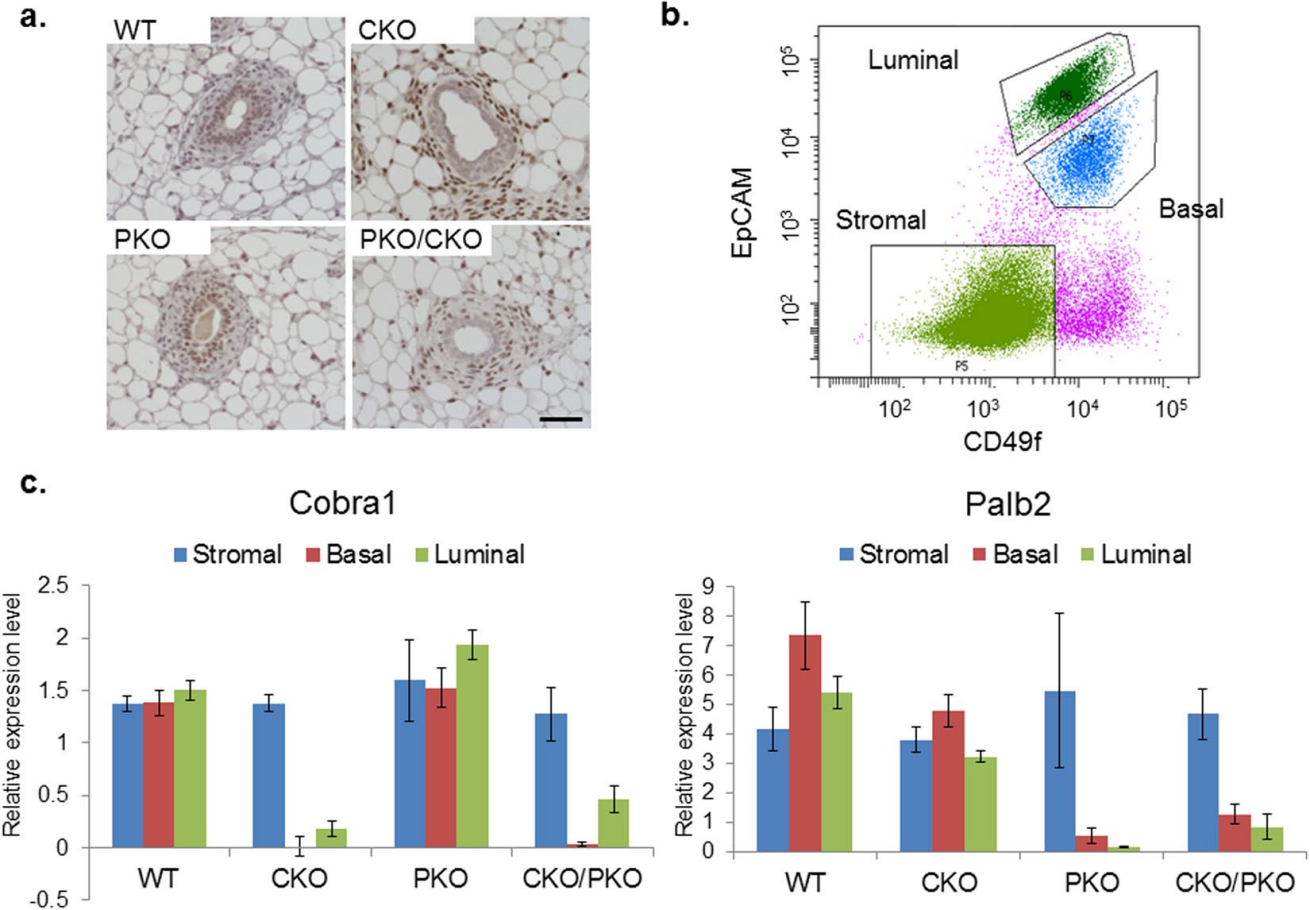
*Palb2* and *Cobra1* are efficiently deleted in mammary epithelium. (**a**) COBRA1 immunohistochemistry analysis in mammary gland of 8-week virgin mice. Representative results from at least 4 sets of animals. Scale bar = 50 µM. (**b**) Representative result of fluorescence-activated cell sorting of mouse mammary glands using cell surface markers EpCAM and CD49f. Cells are sorted to stromal, basal, and luminal populations. (**c**) mRNA analysis of *Cobra1* and *Palb2* using sorted stromal, basal and luminal cells. The numbers of animals used are: WT = 10, CKO = 4, PKO = 4, CKO/PKO = 5. Error bars represent s.e.m.

To address the generality of the genetic interaction between *Brca1* and *Cobra1*, we asked whether other tumor suppressors in the *Brca1* pathway would also display a similar genetic relationship with *Cobra1*. *PALB2* is a breast cancer susceptibility gene and its product interacts with BRCA1^10^. Like BRCA1, PALB2 is involved in HR repair^13,14,39^ and has recently been implicated in transcriptional regulation^40^. We have obtained a previously reported *Palb2*^*f/f*^ mouse strain, in which the *Cre*- mediated recombination resulted in deletion of the coil-coil domain and premature translation termination^38,41^. Due to premature protein truncation, the corresponding RNA transcript is also subjected to degradation via nonsense-mediated decay^38^, resulting in depletion of entire PALB2 protein. We generated mammary epithelial-specific *Palb2* KO (PKO: *Palb2*^*f/f*^; *MMTV-Cre*) and *Cobra1*/*Palb2* double-knockout mice (CKO/PKO: *Cobra1*^*f/f*^ *Palb2*^*f/f*^; *MMTV-Cre*). Immunohistochemistry showed that COBRA1 protein was effectively depleted from mouse mammary epithelium of both CKO and CKO/PKO mice (Fig. 4a). The lack of suitable PALB2-specific antibody precluded us from assessing PALB2 protein levels in WT and mutant mammary glands. Using established cell surface markers, EpCAM and CD49f, we sorted cells from WT and mutant mammary tissue into three populations: stromal cells (EpCAM^−^CD49f^−^), basal epithelial cells (EpCAM^med^CD49f^high^), and luminal epithelial cells (EpCAM^high^CD49f^med^)^42^ (Fig. 4b). Gene expression analysis of sorted cells by real-time PCR (RT-PCR) showed significantly reduced mRNA levels of *Cobra1* and *Palb2* in the basal and luminal compartments, but not the stromal compartment, of CKO/PKO mammary glands (Fig. 4c). These data confirm that *Cre*-mediated recombination results in efficient ablation of these two genes in a cell type-specific manner.

**Figure 5:**
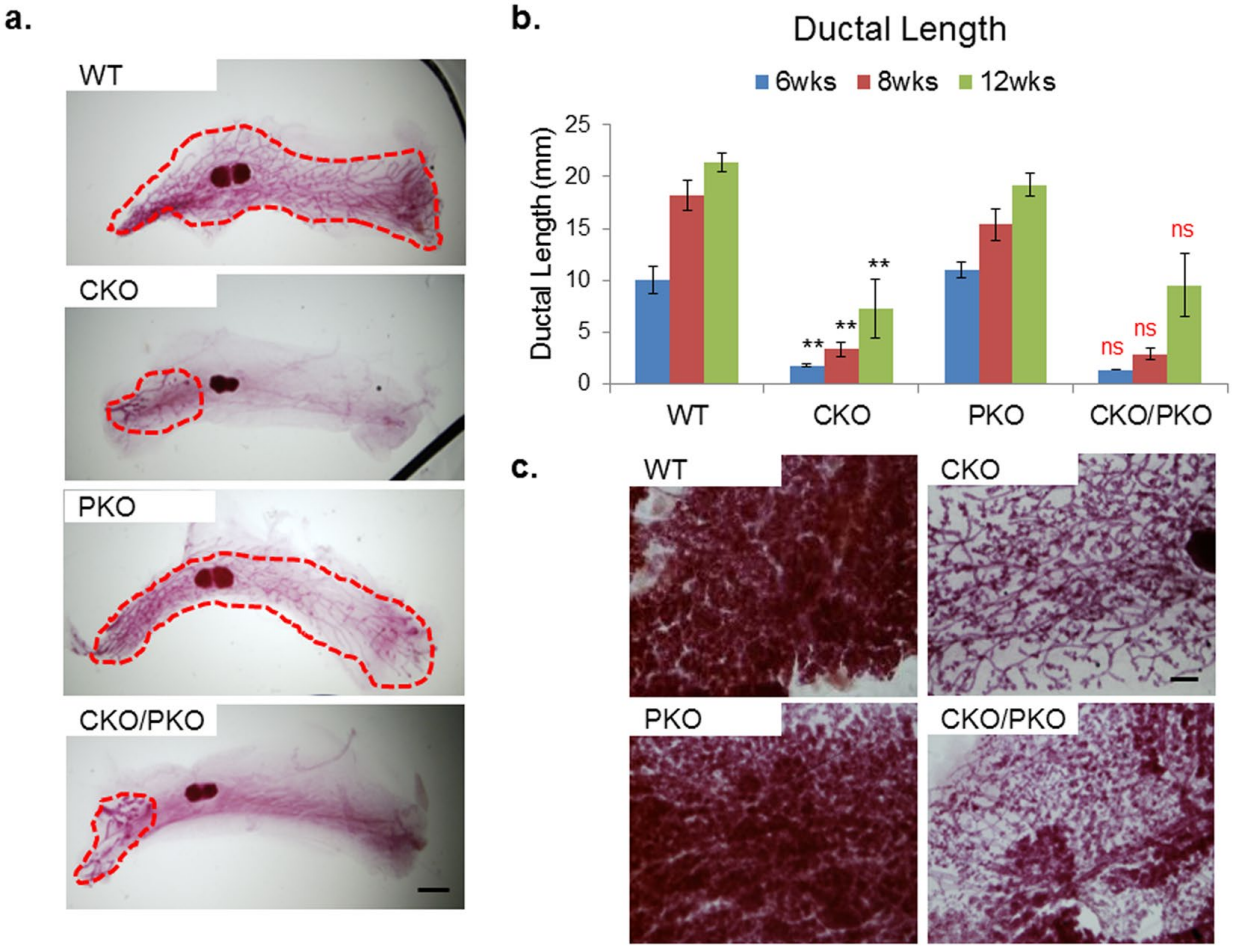
Deletion of *Palb2* did not rescue the developmental defect in CKO mammary glands. (**a**) Whole mounts of mammary glands from 8-week virgin mice. Red dash line highlights the boundary of the ductal area. Images are representatives of at least 4 animals in each group. Scale bars = 1 mm. (**b**) Longitudinal quantification of ductal lengths at 6, 8, and 12-week time points. The numbers of animal used for each of the three time points (6, 8, and 12 wks) are: WT = 4, 4, and 5 mice, CKO = 3, 6, and 5 mice, PKO = 5, 4, and 4 mice, PKO/CKO = 4, 5, 4 mice. Error bars represent s.e.m. Student’s *t*-test was used for statistical analysis comparison between WT and CKO (in black), and between CKO and CKO/PKO (in red). **P < 0.01, ns: not significant. (**c**) Whole mounts of mammary glands from 16 to 20-week mice 1-day postpartum. Scale bar = 500 μm.

Virgin female PKO mice at 8 weeks of age exhibited normal ductal growth, as indicated by epithelial ducts that filled the entire fat pad comparable to WT control (Fig. 5a). However, deletion of *Palb2* did not rescue the ductal growth defect of virgin CKO (Fig. 5a) (see Supplementary Fig. 4a). This is in contrast to our previous observation of the genetic complementation between *Brca1* exon 11 deletion and CKO^37^. Longitudinal quantification of ductal length in mice at 6, 8, and 12 weeks of age indicates that mammary ducts undergo further extension over time in both WT and mutant mammary glands (Fig. 5b). However, mammary ductal development of both CKO and CKO/PKO remained equally retarded as compared to WT and PKO at all three time points examined (Fig. 5b). This data suggest a persistent defect in ductal development in CKO and CKO/PKO mice, rather than a transient delay in ductal growth. To further investigate the functional significance of the ductal growth defects, we also subjected both female WT and mutant mice to mating with WT male mice and analyzed the extent of alveologenesis in dams immediately after pup delivery. Consistent with our previous results^37^, mammary glands of CKO postpartum were largely devoid of alveolar structure (Fig. 5c) (see Supplementary Fig. 4b). Similar to their WT littermates, PKO mice underwent normal alveologenesis with robust alveolar structures and exhibited normal lactating ability. However, CKO/PKO mice displayed a profound alveologenic deficiency (Fig. 5c), resulting in the total inability to nurse (data not shown). In aggregate, these findings underscore the specificity of the genetic interaction between *Cobra1* and *Brca1* in mammary epithelium.

## Discussion

The universality of the extensively characterized DSB repair activity of BRCA1 stands in stark contrast to its tissue-specific tumor suppressor function. In addition to its well-documented role in DSB repair, BRCA1 has also been implicated in other cellular processes including ubiquitination, transcriptional regulation, and heterochromatin-mediated gene silencing^43–46^. Elucidating the biological significance of these diverse BRCA1functions in a physiologically relevant tissue context is pivotal to a better understanding of the molecular basis of BRCA1 function as a tissue-specific tumor suppressor. In the current study, three different *Brca1* mutant mice were used to compare and contrast the allele-specific effects of *Brca1* mutations on mammary epithelial cell-specific *Cobra1* KO. Our data show that only *Brca1* exon 11 deletion (E11^−^), not the I26A or S1598F mutant, is capable of rescuing the mammary developmental defect in CKO mice. This separation-of-function genetic finding supports the notion that the BRCA1 region encoded by exon 11 possesses a particular function of antagonizing the role of COBRA1 in mammary gland development. Given the dedicated role of COBRA1/NELF in Pol II pausing and transcriptional regulation, we propose that this developmental function of BRCA1 is related to transcription of developmentally regulated genes during ductal development. In support, genome-wide analysis indicates that chromatin binding of BRCA1 is enriched at the transcription start sites (TSS) across the human genome^40,47,48^. Furthermore, our published transcriptomic study clearly indicates that *Brca1* exon 11 deletion partially restores the developmentally-related transcription program that is impaired in CKO mammary epithelium^37^. Future work will help uncover the exact biochemical and molecular nature of the functional antagonism between BRCA1 and NELF-dependent Pol II pausing at developmentally important gene loci.

A question related to the current study is whether the genetic interaction between *Brca1* and *Cobra1* is specific to mammary gland. Homozygous deletion of either *Brca1* or *Cobra1* is known to cause early embryonic lethality^49,50^. Breeding of mice that carried hemizygous germ-line deletions of *Brca1* and *Cobra1* (*Brca1*^+/−^, *Cobra1*^+/−^) did not yield any phenotypically normal embryos or viable pups with homozygous deletion of both genes (*Brca1*^*−/−*^, *Cobra1*^*−/−*^), suggesting the lack of genetic complementation during embryogenesis^37^. Future investigation in adult tissues besides mammary glands will help address the question of tissue-specificity of this *Brca1* and *Cobra1* interaction. For example, we have previously reported that *Cobra1* ablation in mouse myocardium led to severe cardiomyopathy^51^. It will be of interest to determine whether *Brca1* deletion alleviates the cardiomyopathy-related phenotypes associated with *Cobra1* gene disruption.

Mammary gland development depends on numerous factors involved in regulation of transcription and signaling events^52–54^. These multiple pathways intertwine to form a complex network that ultimately results in the establishment and homeostasis of a functional mammary gland. It is abundantly clear that a fine balance between normal development and neoplasm is maintained by opposing actions of both positive and negative factors that collectively dictate cell proliferation and differentiation. Mouse genetics provides a powerful tool to dissect the inherent complexity in regulation of mammary development^55,56^. The distinct phenotype of *Cobra1*/*Palb2* (CKO-PKO) versus *Cobra1*/*Brca1* (CKO-E11^−^) mice, which is difficult to predict based on our current knowledge of PALB2 and BRCA1 function in DSB repair, highlights the gene-specific genetic interaction between *Cobra1* and *Brca1*, as well as the power of mouse genetics. Despite extensive efforts, we were not successful in generating compound mutant mice with mammary gland-specific knockout of *Cobra1* and *Brca2* (data not shown), likely due to embryonic lethality associated with the leakiness of the MMTV-Cre system. Nevertheless, it is clear from our *Cobra1*/*Palb2* study that, despite the functional similarity of BRCA1 and PALB2 in DSB repair, these two proteins do not share the ability to antagonize the action of a *bona fide* transcription pausing factor during mammary gland development. This result is also consistent with our earlier finding that the genetic complementation between *Brca1* and *Cobra1* is independent of DSB repair^37^. All in all, our data uncover a domain- and gene-specific functional interaction between *Brca1* and transcriptional pausing factor *Cobra1/Nelf-b* in mammary glands.

## Methods

### Mice

*Cobra1/Nelf-b*^*f/f*^ mice have been described previously^57^. *Palb2*^*f/f*^ (B6;129-*Palb2*^*tm1.1Dli*^) was purchased from the Jackson Laboratory. *Brca1*^*FH-I26A/FH-I26ABrca1FH*^ and *Brca1*^*S1598F/S1598F*^ mice were previously described^35^. *MMTV-Cre* line A mice (from Dr. Anthony Wynshaw-Boris) were used to generate *MMTV-Cre*, *Cobra1*^*f/f*^, *MMTV-Cre*, *Brca1*^*f/f*^, *MMTV-Cre*, *Palb2*^*f/f*^, *MMTV-Cre*, *Brca1*^*f/f*^, *Cobra1*^*f/f*^, *MMTV-Cre*, *Palb2*^*f/f*^, *Cobra1*^*f/f*^, *MMTV-Cre, Brca1*^*FH-I26A/FH-I26ABrca1FH*^, *Cobra1*^*f/f*^ and *MMTV-Cre, Brca1*^*S1598F/S1598F*^, *Cobra1*^*f/f*^ as previously described. The strains used in our genetic study were in a similarly mixed genetic background. Parental *MMTV-Cre* mice, which were used as controls in our published studies, did not show any appreciable defects in mammary gland development^37^. In all experiments, control and mutant littermates were used for comparison. All procedures performed on animals were approved by the Institutional Animal Care and Use Committee (IACUC) at the University of Texas Health San Antonio. All animal experiments were performed in accordance with guidelines and regulations by IACUC at the University of Texas Health San Antonio.

### Whole mount analysis of the mammary glands

Mammary glands from mice of different age groups as indicated were used for whole mount staining. Inguinal mammary glands were isolated and fixed in Carnoy’s fixative (ethanol: chloroform: glacial acetic acid, 60:30:10) overnight at room temperature. They were rehydrated in descending grades of alcohol (70%, 50%, and 30%) for 15 min each, then rinsed with distilled water before putting in Carmine alum for overnight staining. Stained glands were dehydrated in ascending grades of alcohol (70% twice, 90%, 95%, and 100% twice) for 15 min each, and cleared with Citrisolv reagent (Fisher, Cat#. 22-143975). Glands were mounted and examined under a Nikon SMZ1000 dissection microscope. Eclipse software was used to measure ductal length of calibrated image. Average length of three longest ducts from nipple region was used to represent the ductal length of each animal.

### Immunohistochemistry (IHC) staining

Mammary glands were fixed in 10% Neutral buffered formalin for 16–18 hr at 4 °C and paraffin embedded. 3 µM paraffin section slides were first de-paraffinized with xylene, and then rehydrated in descending grade of alcohol (100%, 95%, 70%, and 50%). Samples were washed briefly with PBS before transferring to boiling antigen-unmasking solution (Vector Labs, H-3300) for 20 min. Endogenous peroxidase was blocked by pre-incubating slides in 3% hydrogen peroxide for 10 min followed by 10% normal goat serum in PBS for 1 hr blocking at room temperature. Primary antibody (anti-NELF-B/COBRA1, 1:50) was added and incubated overnight at 4 °C. For detection with primary antibody using the immune enzymatic method, the ABC peroxidase detection system (Vector Labs, PK-6105) was used with 3,3′-diaminobenzidine (DAB) as substrate (Vector Labs, SK-4105) according to manufacturer’s instruction.

### Primary mammary epithelial cell (MEC) isolation and fluorescence-activated cell sorting (FACS)

Fresh mammary glands (3rd, 4^th^, and 5^th^ pairs) from 8–10 week virgin mice were used to isolate primary MEC. Single cells were prepared using published protocol^58^ with minor modifications. All reagents were purchased from StemCell Technologies (Vancouver, Canada), unless otherwise indicated. Briefly, isolated glands were minced and digested in dissociation solution containing 1 mg/mL collagenase and 100 U/ml hyaluronidase (Cat# 07919), 2% FBS, insulin (5 mg/ml), penicillin-streptomycin and DMEM-F12 for 15–18 hr at 37 °C with gentle rocking. After overnight treatment, epithelial pellets were collected and lysed with 0.8% NH_4_Cl to remove red blood cells (RBCs). The resulting epithelial organoids were subjected to a serial enzymatic digestion with 0.05% pre-warmed trypsin (Life Technologies, 25300) and 5 mg/ml Dispase (Cat# 07913) with 0.1 mg/ml DNase I (Sigma-Aldrich, D4513) before filtering through a 40-µm cell strainer (Fisher, Cat# 22363547) to obtain single cell suspension. Cells were counted, resuspended in ice-cold Hanks Balanced Salt Solution (Cat# 37150) with 2% FBS (HF), and blocked for 10 min on ice with 10% rat serum (Jackson Laboratories, Cat# 012-000-120). After blocking, cells were incubated for 20 min with antibodies for the following cell-surface markers: Ep-CAM-PE (BioLegend, Cat# 118206), CD49f-FITC (BD Biosciences, Cat# 555735), CD31-Biotin (BD Bioscience, Cat# 553371), CD45 biotin (BioLegend, Cat# 103103), TER-119 Biotin (BioLegend, Cat# 103511) followed by Streptavidin-Pacific Blue (Invitrogen, Cat# S11222) incubation. 7-AAD (BD Biosciences, Cat# 559925) was added 10 min before analysis. Sorting was performed with a BD FACSAria flow cytometer (Beckmen Coulter). Data were analyzed using a FACSDiva software.

### Quantitative RT-PCR

Total RNA was isolated using RNeasy Micro kit (Qiagen, Cat# 217004) from sorted cells and used for random hexamer-based reverse-transcription (ImProm-II™ Reverse Transcription System, Promega, Cat# A3800) according to the manufacturer’s instructions. qRT-PCR was performed in an ABI-7300 sequence detection system (Applied Biosystems) using HiGreen High ROX qPCR Master Mix (Thermo Scientific, Cat# K0364). Each measurement was performed in duplicate and expression levels of β-actin were used to normalize the amount of the investigated transcript. The following primers were used for quantitative RT-PCR (F, forward; R, reverse): β-Actin-F: 5′-CGGTTCCGATGCCCTGAGGCTCTT-3′, β-Actin-R: 5′-CGTCACACTTCATGATGGAATTGA-3′ Cobra1-F: 5′-ACAACTTCTTCAGCCCTTCCC-3′, Cobra1-R: 5′-TCTGCACCACCTCTCCTTGG-3′, Palb2-F: 5′-CCTCAGCTATGCGGAGAAGG-3′, Palb2-R: 5′-CTTTTGGCACGCTGAAGTCG-3′.

### Genotyping

The following primers were used for *Cobra1, Brca1-I26A, Brca1-S1598F*, and *MMTV-Cre* genotyping. Cobra1-F: 5-AGACACCCCTCACCCACTCTT-3′, Cobra1-R1:5′-AAGCATCCCTGATCCTCAGGT-3′, Cobra1-R2: 5′-TGTGGGCATGCTGTAGACACA-3′, where F/R1 were paired for detection of the wild-type and floxed alleles, and F/R2 for the null allele. I26A-F: 5′-GGGAAAGAAAGTTGGCAAGG-3′, I26A-R: 5′-CTGGACAGGGAGGAGGGATG-3′, S1598F-F1: 5′-CCCTTGTGCACCTCCAGAGA-3′, S1598F-F2: 5′-GACCTGCAGCCCAAGCTAGC-3′, S1598F-R: 5′-GCCACGCCTATGAAGGCTCT-3′. The MMTV-Cre transgene was genotyped with primers Cre-F: 5′-ACCAGCCAGCTATCAACTCG-3′, Cre-R: 5′-TTACATTGGTCCAGCCACCC -3′, yielding an ∼300-bp band in MMTV-Cre transgenic animals. Ctrl-F: 5′-CTAGGCCACAGAATTGAAAGATCT -3′ and Ctrl-R: 5′-GTAGGTGGAAATTCTAGCATCATCC-3′ were used as internal control.

### Data availability

No datasets were generated or analyzed during the current study.

## Electronic supplementary material

supplemental information
