# Supplementary material for: Gene-Specific Genetic Complementation between Brca1 and Cobra1 During Mouse Mammary Gland Development
Source: Sci Rep. 2018 Feb 9;8:2731. doi: 10.1038/s41598-018-21044-2 (PMC5807304; doi:10.1038/s41598-018-21044-2)
Supplement: Supplementary file 1 — supplemental information [file 41598_2018_21044_MOESM1_ESM.pdf]

Supplemental Information

Title: Gene-Specific Genetic Complementation between *Brca1* and *Cobra1* During Mouse Mammary Gland Development

Authors: Huai-Chin Chiang[1], Xiaowen Zhang[1], Xiayan Zhao[2], Chi Zhang[1], Jerry Chen[1], Paula Garza[1], Sabrina Smith[1], Thomas Ludwig[3], Richard J. Baer[4], Rong Li[1*], Yanfen Hu[1*]

[1]Department of Molecular Medicine, University of Texas Health San Antonio San Antonio, TX 78229, USA

[2]Xiangya School of Medicine, Central South University, Changsha, Hunan, China

[3]Department of Cancer Biology and Genetics, Ohio State University, Columbus, OH 43210, USA

[4]Department of Pathology & Cell Biology, New York, NY 10032, USA

[*]Co-Corresponding Authors

Department of Molecular Medicine

University of Texas Health Science Center at San Antonio

8403 Floyd Curl Drive

STRF, Room 219

San Antonio, TX 78229

Office phone: 210-562-4152 (R.L.); 210-562-4153 (Y.H.)

Email: lir3@uthscsa.edu (editorial communication), huy3@uthscsa.edu

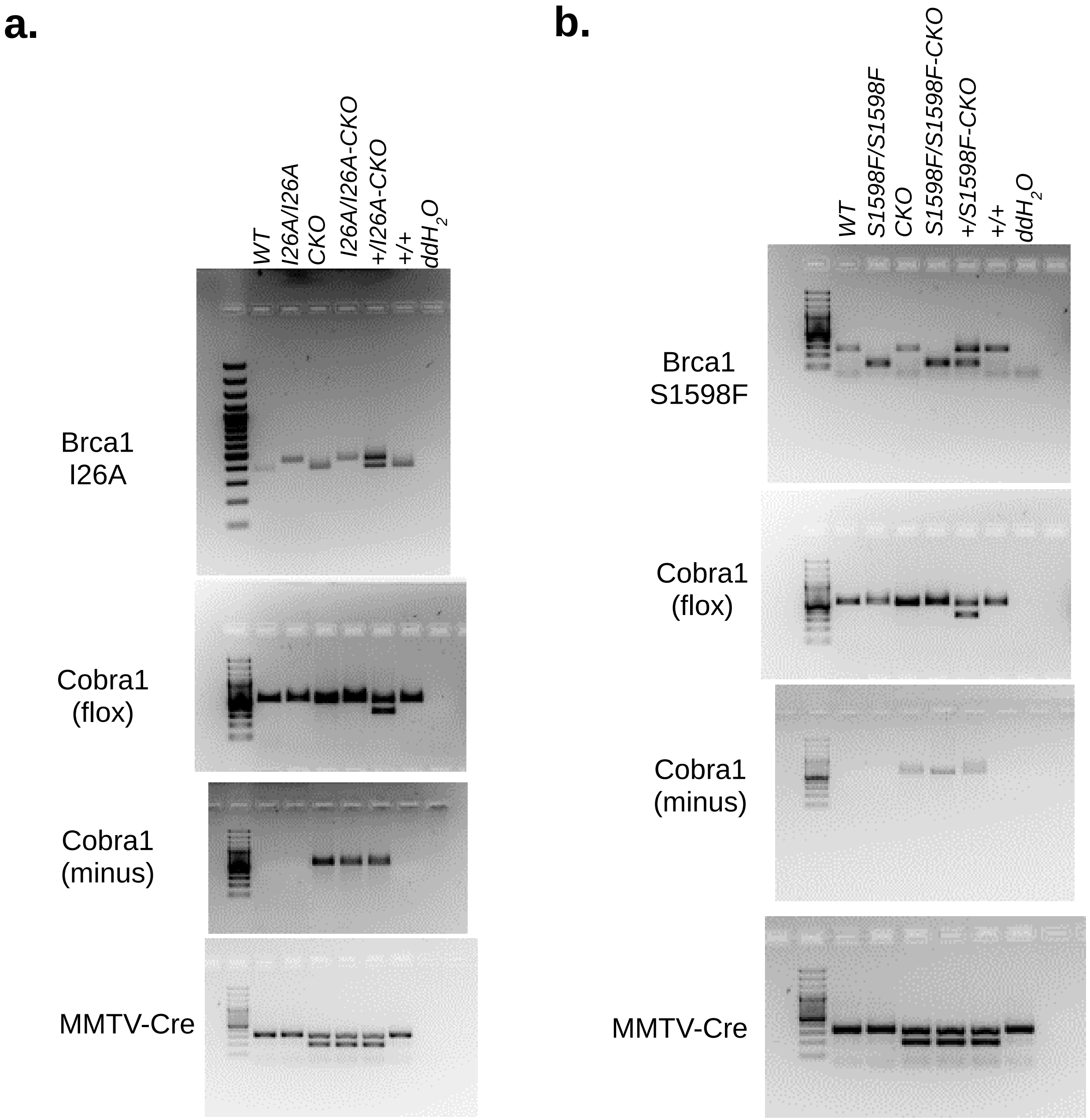

Supplementary Figure 1. Full-length gels of genotyping analysis for the mutant mice. (a) Representative genotyping results of CKO-I26A compound mice. **(b)** Representative genotyping results of CKO-S1598F compound mice.

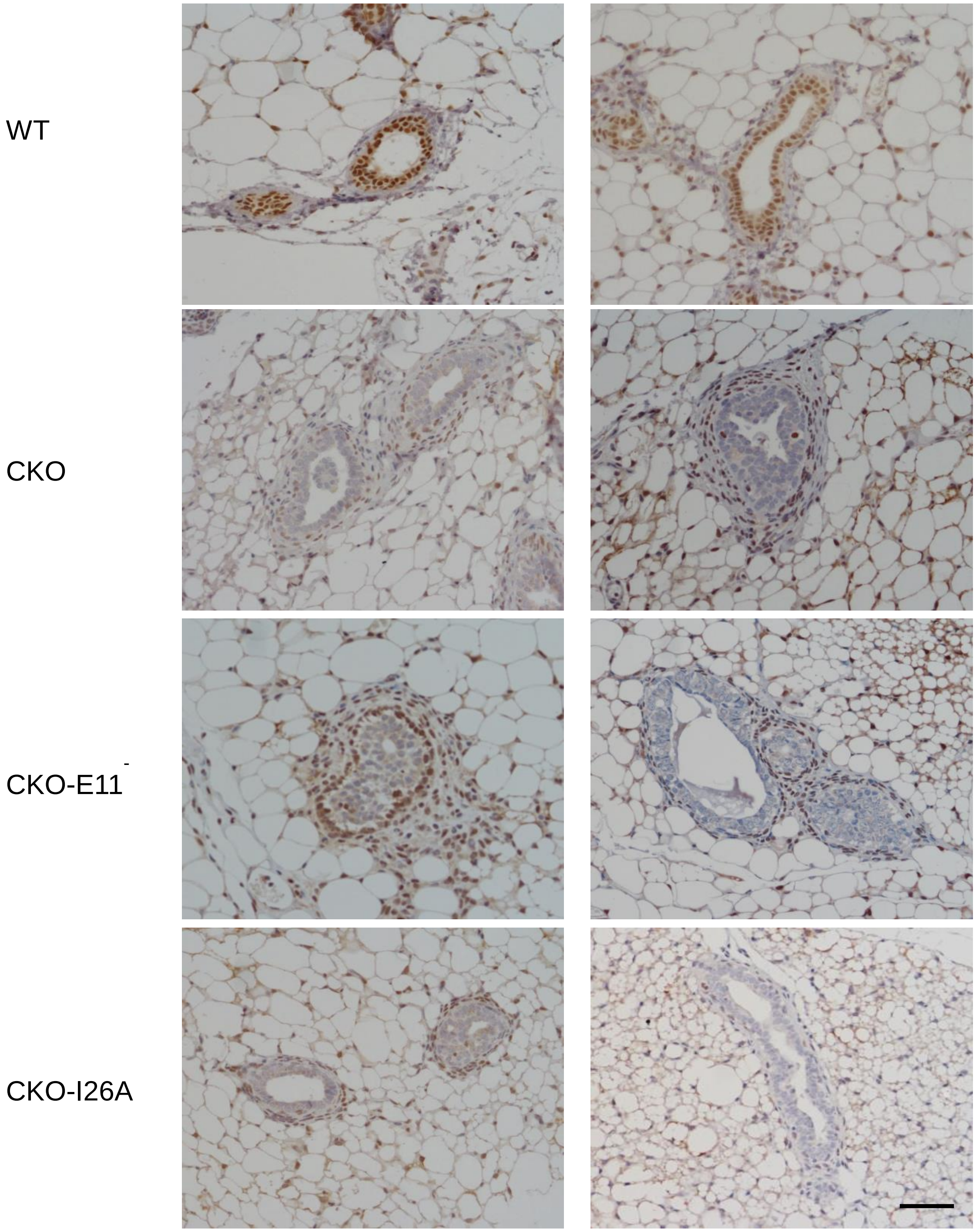

Supplementary Figure 2. Additional images of COBRA1 immunohistochemistry analysis in mammary gland of different 8-week virgin mice. Scale bar =50μM.

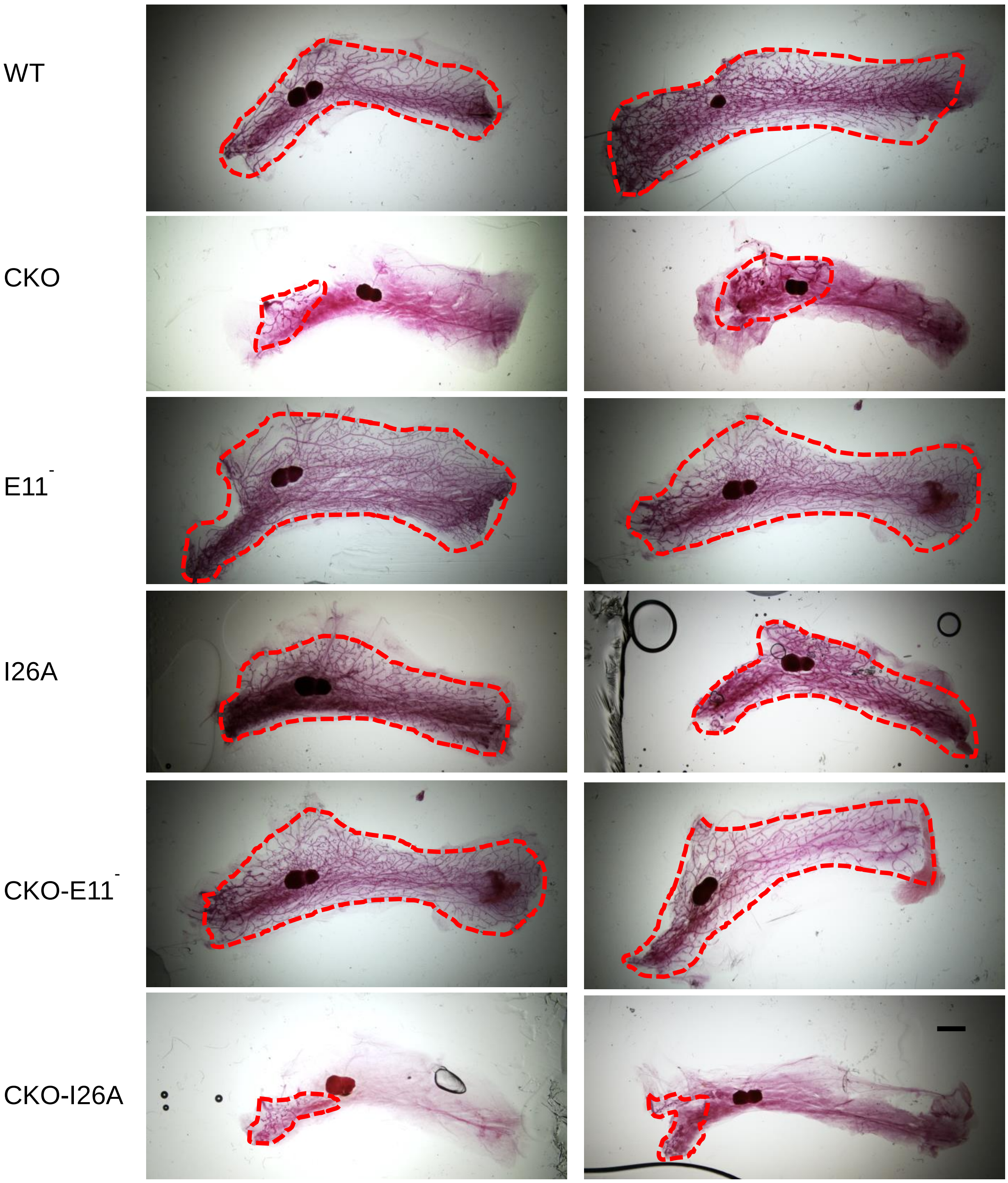

Supplementary Figure 3. Additional whole mount images from different 8-week virgin mice.

Red dash line highlights the boundary of the ductal area. Scale bar =2mm.

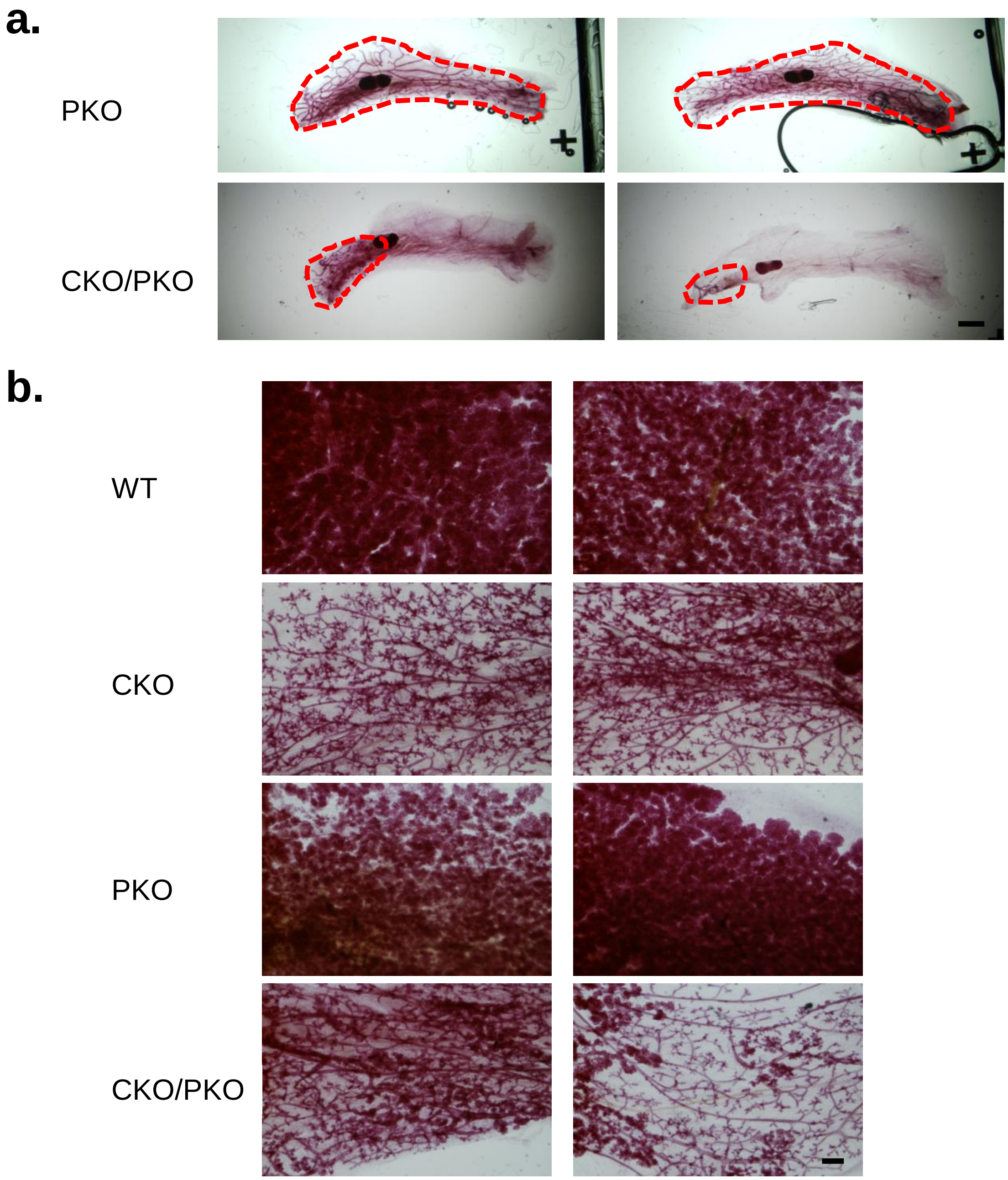

Supplementary Figure 4. Additional whole mount images from different mice. (a) Whole mounts of mammary glands from 8-week virgin mice. Red dash line highlights the boundary of the ductal area. Scale bar =2mm. **(b)** Whole mounts of mammary glands from 16 to 20-week mice 1-day postpartum. Scale bar=500 μm.
